# A Penicillin Derivative Exerts an Anti-Metastatic Activity in Melanoma Cells Through the Downregulation of Integrin αvβ3 and Wnt/β-Catenin Pathway

**DOI:** 10.3389/fphar.2020.00127

**Published:** 2020-02-25

**Authors:** Elizabeth Barrionuevo, Florencia Cayrol, Graciela A. Cremaschi, Patricia G. Cornier, Dora B. Boggián, Carina M. L. Delpiccolo, Ernesto G. Mata, Leonor P. Roguin, Viviana C. Blank

**Affiliations:** ^1^Laboratorio de Oncología y Transducción de Señales, Instituto de Química y Fisicoquímica Biológicas (IQUIFIB), Departamento de Química Biológica, Facultad de Farmacia y Bioquímica, Universidad de Buenos Aires, CONICET, Buenos Aires, Argentina; ^2^Laboratorio de Neuroinmunomodulación y Oncología Molecular, Instituto de Investigaciones Biomédicas, Facultad de Ciencias Médicas, Pontificia Universidad Católica Argentina (UCA), CONICET, Buenos Aires, Argentina; ^3^Laboratorio de Química Orgánica, Instituto de Química Rosario (CONICET-UNR), Facultad de Ciencias Bioquímicas y Farmacéuticas, Universidad Nacional de Rosario, Rosario, Argentina

**Keywords:** triazolylpeptidyl penicillin, anti-metastatic effect, murine melanoma, β-catenin, metalloproteinases-2 and -9, integrin αvβ3

## Abstract

The synthetic triazolylpeptidyl penicillin derivative, named TAP7f, has been previously characterized as an effective antitumor agent *in vitro* and *in vivo* against B16-F0 melanoma cells. In this study, we investigated the anti-metastatic potential of this compound on highly metastatic murine B16-F10 and human A375 melanoma cells. We found that TAP7f inhibited cell adhesion, migration and invasion in a dose-dependent manner. Additionally, we demonstrated that TAP7f downregulated integrin αvβ3 expression and Wnt/β-catenin pathway, a signaling cascade commonly related to tumor invasion and metastasis. Thus, TAP7f reduced both the enzymatic activity and the expression levels of matrix-metalloproteinases-2 and -9 in a time dependent manner. Moreover, TAP7f inhibited the expression of the transcription factor Snail and the mesenchymal markers vimentin, and N-cadherin, and up-regulated the expression of the epithelial marker E-cadherin, suggesting that the penicillin derivative affects epithelial–mesenchymal transition. Results obtained *in vitro* were supported by those obtained in a B16-F10-bearing mice metastatic model, that showed a significant TAP7f inhibition of lung metastasis. These findings suggest the potential of TAP7f as a chemotherapeutic agent for the treatment of metastatic melanoma.

## Introduction

Metastasis is a sequential multistep process that enables the physical translocation of cancer cells from primary tumors to distant tissues. This process is the cause of the majority of cancer deaths, not only as a result of the invasion of vital tissues but also because of the harmful side effects of chemotherapeutic drugs employed for its treatment (Steeg, 2016; Lambert et al., 2017). The first step in metastasis is the escape of tumor cells from the primary tumor after epithelial to mesenchymal transition (EMT), a conversion in cell phenotype by which cancer cells acquire migratory and invasive capacities (Thiery, 2002; Lamouille et al., 2014). One of the signaling pathways that has been implicated in EMT is the Wnt/β-catenin signaling pathway (Ghahhari and Babashah, 2015; Kovacs et al., 2016). The essential component of this pathway, β-catenin, interacts with T-cell factor/lymphoid enhancing factor (TCF/LEF) to activate the transcription of multiple target genes implicated in metastasis (Valenta et al., 2012; Zhan et al., 2017). Extracellular matrix (ECM) degradation is a fundamental process that promotes tumor metastasis and is produced by a family of zinc-dependent endopeptidases called matrix metalloproteinases (MMPs) (Visse and Nagase, 2003; Jabłońska-Trypuć et al., 2016). Due to MMPs enzymatic activity, tumor cells can migrate to their secondary sites of growth *via* blood and lymphatic vessels. MMP-2 (also known as Gelatinase A) and MMP-9 (also known as Gelatinase B) are highly expressed and activated in many human tumors (Egeblad and Werb, 2002; Mehner et al., 2014). Furthermore, it has been demonstrated that Wnt/β-catenin signaling influence the expression of MMP-2 and MMP-9 (Qu et al., 2014). Other proteins that play a role in metastatic dissemination are integrins, heterodimeric transmembrane proteins that facilitate interactions between cells and the ECM and are involved in cell proliferation, differentiation, adhesion and migration (Hynes, 2002; Luo and Springer, 2006).

Melanoma is a very aggressive form of skin cancer that has an important incidence of mortality because it is highly metastatic (Domingues et al., 2018). Therefore, the finding of new compounds that can inhibit melanoma metastasis is crucial and a main challenge to be solved. In a previous work, we have obtained and characterized a series of penicillin derivatives (triazolylaminoacyl(peptidyl) penicillins: TAPs) that were obtained by conjugation of penicillin to different aminoacids or dipeptides *via* a triazole group (Cornier et al, 2014). The triazolylpeptidyl penicillin derivative TAP7f, with leucine and phenylalanine bound to the triazole group, was the most potent and selective TAP tested, showing 30 times more antiproliferative activity on tumor cells than on normal cells (Cornier et al, 2014). We have also demonstrated that TAP7f induced an antitumor effect through the induction of cell cycle arrest and the activation of both death receptor and mitochondria-dependent apoptotic pathways in melanoma B16-F0 cells (Blank et al., 2018). Moreover, when TAP7f was evaluated *in vivo* in a B16-F0 murine melanoma model, results showed a reduction of approximately 70% of tumor growth (Blank et al., 2018). In order to continue exploring TAP7f biological properties, we herein investigated the *in vitro* anti-metastatic effect of this compound in murine B16-F10 and human A375 melanoma cells. Additionally we explored TAP7f effect in a B16-F10-bearing mice experimental metastasis model.

## Materials and Methods

### Reagents and Antibodies

**Figure 1:**
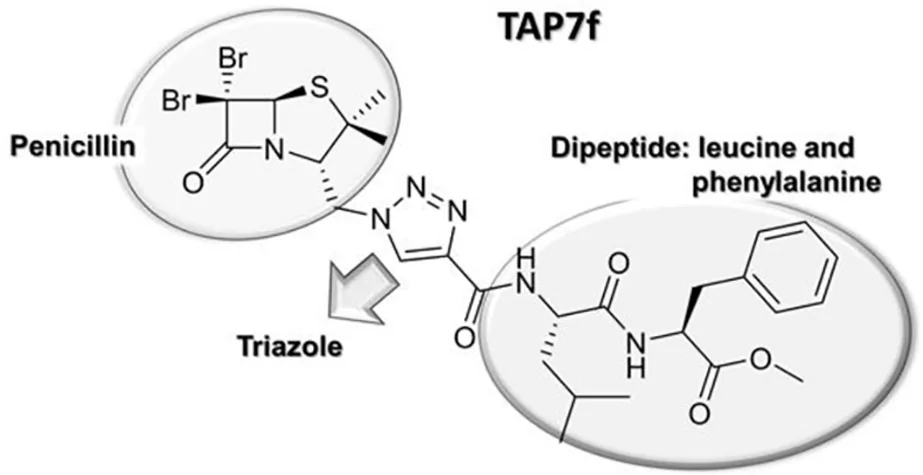
TAP7f chemical structure.

TAP7f was synthesized as described in a previous work (**Figure 1**, Cornier et al, 2014). A 100 mM stock solution of the compound was prepared in dimethyl sulfoxide (DMSO) and stored at −70°C. The stock solution diluted 1/10 in ethanol was used for *in vitro* assays at different concentrations in the indicated culture medium. All the experiments were performed with a final concentration of 20 µl vehicle/ml of medium. Antibodies for MMP-2, MMP-9, c-Myc, cyclin-D1, E-cadherin, N-cadherin, Snail and vimentin were purchased from Santa Cruz Biotechnology Inc (Dallas, TX, USA). Rabbit monoclonal antibody anti-β-catenin was from Cell Signaling Technology (Danvers, MA, USA). Geltrex™ Reduced Growth Factor Basement Membrane Matrix was from Thermo Fisher Scientific (Waltham, MA, USA).

### Cell Lines and Culture Conditions

B16-F10 cells (murine melanoma, ATCC CRL-6475) and M1/15 cells (derived from liver metastasis developed in immunosuppressed mice inoculated with a human melanoma cell line, gently supplied by Dr. Andras Falus, Department of Genetics, Cell and Immunobiology, Semnelweiss University, School of Medicine, Hungary) were grown in RPMI-1640 medium (Gibco BRL, USA) supplemented with 10% fetal bovine serum (FBS), 2 mM L-glutamine, 50 U/ml penicillin and 50 µg/ml streptomycin. A375 cells (human malignant melanoma, ATCC CRL-1619) were grown in DMEM-F12 medium supplemented with 10% fetal bovine serum (FBS), 2 mM L-glutamine, 50 U/ml penicillin and 50 µg/ml streptomycin. MDA-MB-231 cells (human mammary gland adenocarcinoma, ATCC HTB-26) were grown in DMEM with 4.5 g/L glucosa containing 10% FBS, 2 mM L-glutamine, 50 U/ml penicillin, and 50 μg/ml streptomycin. F3II cells (murine mammary adenocarcinoma, generously provided by the Laboratory of Molecular Oncology, Quilmes National University, Buenos Aires, Argentina) were grown in minimum essential medium supplemented with 10% FBS, 2 mM L-glutamine, 50 U/ml penicillin, and 50 μg/ml streptomycin. SK-MEL-28 cells (human malignant melanoma, ATCC HTB-72) and JEG-3 cells (human choriocarcinoma, ATCC HTB-36) were maintained in the same conditions, but adding 1 mM sodium pyruvate, 4 mM sodium bicarbonate and 1 mM nonessential amino acids.

### Proliferation Assay

Cells were incubated in 96-well microplates at a density of 1 × 10^4^ cells/well (B16-F10, M1/15, F3II, A375 and MDA-MB-231) or 5 × 10^3^ cells/well (JEG-3 and SK-MEL-28), for 72 h at 37°C in the presence of different concentrations of TAP7f or vehicle, in a total volume of 0.2 ml of the corresponding culture medium. Cell number was evaluated by colorimetric quantification of the levels of the ubiquitous lysosomal enzyme hexosaminidase (Landegren, 1984).

### Adhesion Assay

B16-F10 cells were incubated with different concentrations of TAP7f or vehicle for 18 h. Afterwards, cells were trypsinized, counted and seeded into 96-well microplates (40,000 cells/well) pre-coated for 18 h with fibronectin (1 µg/ml) or vitronectin (1 µg/ml). After 1 h, unattached cells were washed three times with PBS and attached cells were detected by colorimetric quantification of the levels of hexosaminidase.

### Wound Healing Assay

B16-F10 or A375 cells were plated on 96-well microplates and grown to 90% confluence in 100 µl of complete culture medium. Cell monolayers were scratched using a p10 pipette tip and then treated with different concentrations of TAP7f. Cells were allowed to migrate for 48 h and photographs were taken with an inverted microscope at different times. The wound area was analyzed using Image J software.

### Invasion Assay

Invasion assay was performed by using Geltrex™ coated Boyden chambers with 8 μm pore filter inserts in 24-well plates (BD Biosciences, NY, USA). Briefly, 1 × 10^6^ cells/ml in culture medium without FBS were added to the inserts and 10% FBS supplemented media was added to the bottom well. Treatments were added to upper well chambers according to the experimental requirement. After 18 h, the non-invading cells were gently removed with cotton swabs; invading cells were first fixed in 4% formaldehyde and then stained with Crystal violet, air-dried, counted and photographed.

### Gelatin Zymography

The presence of MMPs in supernatants obtained after incubating cells with or without TAP7f was detected by gelatin zymography. The harvested culture media were diluted 1:2 with non-reducing sample buffer (0.063 M Tris/HCl, pH 6.8, 2% SDS, 10% glycerol, 0.05% bromophenol blue) and then submitted to electrophoresis using 10% acrylamide gels containing 2 mg/ml gelatin type B (Sigma, St. Louis, MO, USA). After electrophoresis, proteins were renatured by incubating gels for 1 h at room temperature with 10 mM Tris, 130 mM NaCl (TBS), pH 7.4, containing 2.5% Triton X-100 to remove SDS, then for 15 min in TBS and, afterwards, gels were incubated for 22 h at 37°C in 120 mM NaCl, 5 mM CaCl2, 1 mM ZnCl2, 50 mM Tris–HCl, pH 7.5. Finally, gels were stained for 30 min with 0.25% Coomasie Brilliant Blue R-250 (Sigma, St. Louis, MO, USA) in methanol: acetic acid: water (4:1:5). Gelatinolytic activities were detected as clear bands on a uniformed blue background. For quantification of band intensities, gels were scanned using a densitometer (Gel Pro Analyzer 4.0, Meyer Instruments. Houston, TX, USA).

### Western Blot

B16-F10 or A375 cells (1.5 × 10^6^) were incubated for different times in the presence of 10 µM of TAP7f or vehicle in 6-well microplates, harvested and washed with cold PBS. Then, 1 × 10^6^ cells were lysed for 30 min at 4°C in 10 µl of lysis buffer (10% glycerol, 0.5% Triton X-100, 1 μg/ml aprotinin, 1 μg/ml trypsin inhibitor, 1 μg/ml leupeptin, 10 mM Na_4_P_2_O_7_, 10 mM NaF, 1 mM Na_3_VO_4_, 1 mM EDTA, 1 mM PMSF, 150 mM NaCl, 50 mM Tris, pH 7.4). Cell lysates were centrifuged and aliquots of supernatants containing 100 μg of protein were resuspended in 0.063 M Tris/HCl, pH 6.8, 2% SDS, 10% glycerol, 0.05% bromophenol blue, 5% 2-mercaptoethanol, submitted to SDS-PAGE and transferred onto PVDF membranes (GE Healthcare, Chicago, IL, USA) for 1 h at 100V in 25 mM Tris, 195 mM glycine, 20% methanol, pH 8.2. Membranes were then treated as the usual western blotting method. The secondary antibodies were anti-mouse IgG (horseradish peroxidase-conjugated goat IgG from Santa Cruz Biotechnology, sc-2005) or anti-rabbit IgG (horseradish peroxidase-conjugated goat IgG from Santa Cruz Biotechnology, sc-2004). Immunoreactive proteins were visualized using the Pierce^®^ ECL Plus detection system (Thermo Fisher Scientific, Waltham, MA, USA) according to the manufacturer’s instructions. Band intensity was quantified by using a densitometer (Gel Pro Analyzer 4.0). Mouse anti-tubulin antibody (Abcam, Cambridge, UK) was used to confirm equal protein loading.

### Reverse Transcription Polymerase Chain Reaction

Cell samples were homogenized in Tri-Reagent (AP biotech, Buenos Aires, Argentina) and total RNA was isolated following the manufacturer’s instructions. RNA pellets were dissolved in RNase-free water and stored at −80°C. The RNA concentration was quantified by measuring the absorbance at 260 nm. The samples were used for RT–PCR analysis. Complementary DNA (cDNA) was synthesized by retrotranscription using the M-MLV reverse transcriptase (Promega, Madison, WI; USA). cDNA amounts present in each sample were determined using a commercial master mix for Real-Time PCR containing SYBR Green fluorescent dye (TransStart^®^ Green qPCR SuperMix, AP biotech, Argentina). qPCR reactions were carried out in an Applied Biosystems 7500. The primer sequences (Biodynamics SRL, Argentina), were designed using Primer Express software version 3.0 (Applied Biosystems, Foster City, CA, USA) with a melting temperature of 60–61°C. Quantification of the target gene expression was performed using the comparative cycle threshold (Ct) method according to the manufacturer’s instructions (Applied Biosystems). An average Ct was obtained from the duplicate reactions and normalized to β2-microglobulin and the ΔΔCt was calculated.

### Flow Cytometry Assay

Expression of integrins receptors was analyzed by staining B16-F10 cells with anti- integrin αV and anti-integrin β3 antibodies (sc-376156 and sc-365679, respectively, Santa Cruz Biotechnology, Dallas, TX, USA) and the corresponding isotype control antibody (BD Pharmingen™, San Diego, CA, USA). Prior to incubation with the primary antibody, cells were fixed in paraformaldehyde 1% and washed. After overnight incubation at 4°C with the primary antibody, the cells were washed and incubated with the corresponding conjugated secondary antibody, anti-mouse-PE (cat:550589, BD Pharmingen™). At least 10,000 cells were acquired using a BD Accuri™ C6 flow cytometer.

### Immunofluorescence

B16-F10 or A375 melanoma cells grown up on coverslips were incubated or not for 18 h with TAP7f 10 µM, washed with PBS and fixed for 5 min at room temperature with 4% paraformaldehyde. After washing with PBS, monolayers were incubated with PBS, BSA 1% for 2 h at room temperature to block non-specific binding sites. Then, coverslips were incubated overnight at 4°C with rabbit antibody anti- β-catenin or mouse antibody anti- E-cadherin diluted in PBS, BSA 1%. In parallel, normal mouse IgG was employed as negative control. After rinsing with PBS, coverslips were incubated for 2 h at room temperature with anti-rabbit Cy3-conjugated secondary antibody (Abcam, Cambridge, UK) or anti-mouse FITC-conjugated secondary antibody (Santa Cruz Biotechnology, Dallas, TX, USA). Nuclear counterstaining was performed by adding the blue-fluorescent nucleic acid stain Höechst 33258 (Sigma, St. Louis, MO, USA) to the anti-fade mounting medium. Cells were examined with an Olympus BX50 epifluorescence microscope provided with a Cool-Snap digital camera. Fluorescence intensity quantification was performed employing Image J software.

### *In Vivo* Anti-Metastatic Effect of TAP7f

*In vivo* experiments were carried out in accordance with the principles of the Basel Declaration and recommendations of the National Institute of Health (NIH) Guide for the Care and the Use of Laboratory Animals, and approved by the Institutional Animal Care and Use Committee (CICUAL) of the School of Pharmacy and Biochemistry, University of Buenos Aires. Female C57BL/6J mice, obtained from the Animal Care Facility of the School of Veterinary, University of Buenos Aires, were housed under controlled conditions and were routinely used at 12–14 weeks old (approximate weight: 20–25 g). Food and water were administered *ad libitum*. To study the metastatic capacity of TAP7f pre-treated B16-F10 cells, cells were incubated with TAP7f or vehicle for 18 h, cell viability was checked by Trypan Blue dye exclusion test and equal number of cells (400,000/100 μl RPMI) were injected in C57BL/6J mice through the tail vein. Nine days after cell inoculation, animals were sacrificed and lungs were excised, photographed, fixed in formaldehyde buffer 10% in PBS 0.1 M, pH 7.4, and then dehydrated and included in paraffin. Cuts of 5 µm were made in microtome (Leica RM 2125. Wetzlar, Germany) and mounted on 2% xylane-coated slides. Sections were then stained with hematoxylin–eosin for histological analysis. To study the anti-metastatic effect of TAP7f, B16-F10 cells (300,000/100 μl RPMI) were injected in C57BL/6J mice through the tail vein. Following the cell injection, mice were intraperitoneally injected daily with 10 mg/kg of TAP7f or vehicle (70% (v/v) polyethylene glycol 400 in PBS). All mice were sacrificed at 12 days after tumor injection. Lungs were removed and fixed. Metastastic foci at lung surfaces were photographed and counted.

### Statistical Analysis

All values are expressed as mean ± SE, p <0.05 was considered statistically significant. Analyses were performed using GraphPad Prism 5.00 software. Statistical analysis of data was performed by using the Student’s t-test or one-way ANOVA followed by Dunnett’s multiple comparison tests.

## Results

### TAP7f Inhibits Proliferation of Different Metastatic Cells

We have previously reported that TAP7f induces an antiproliferative activity on different tumor cell lines (Cornier et al, 2014; Blank et al., 2018). In this work, we explored TAP7f effect on the proliferation of metastatic cells from different sources (**Table 1**). IC_50_ values obtained showed that melanoma B16-F10 and A375 cells, mammary adenocarcinoma MDA-MB-231 and choriocarcinoma JEG-3 cells were the most vulnerable to the penicillin derivative antiproliferative activity with IC_50_ values around 10 µM. In order to evaluate the *in vitro* effect of TAP7f on cell functions that are usually involved in metastasis, we further choose murine B16-F10 and human A375 melanoma cells. Additionally, we studied TAP7f effect in a B16-F10 murine model of experimental metastasis.

**Table 1:** IC_50_ values of TAP7f on different metastatic human and murine cell lines.

Cell lines		IC_50_ (μM)^a^
Human	SK-MEL28	17 ± 3
	JEG-3	11 ± 2
	MDA-MB-231	13 ± 4
	M1/15	18 ± 2
	A375	10 ± 1
Murine	B16-F10	7 ± 2
	F3II	16 ± 2

a The molar concentration required to cause 50% growth inhibition (IC_50_) was determined from dose–response curves. Results represent the mean ± S.E. of at least three different experiments.

### TAP7f Reduces Cell Adhesion, Migration and Invasion of Metastatic Melanoma Cells

**Figure 2:**
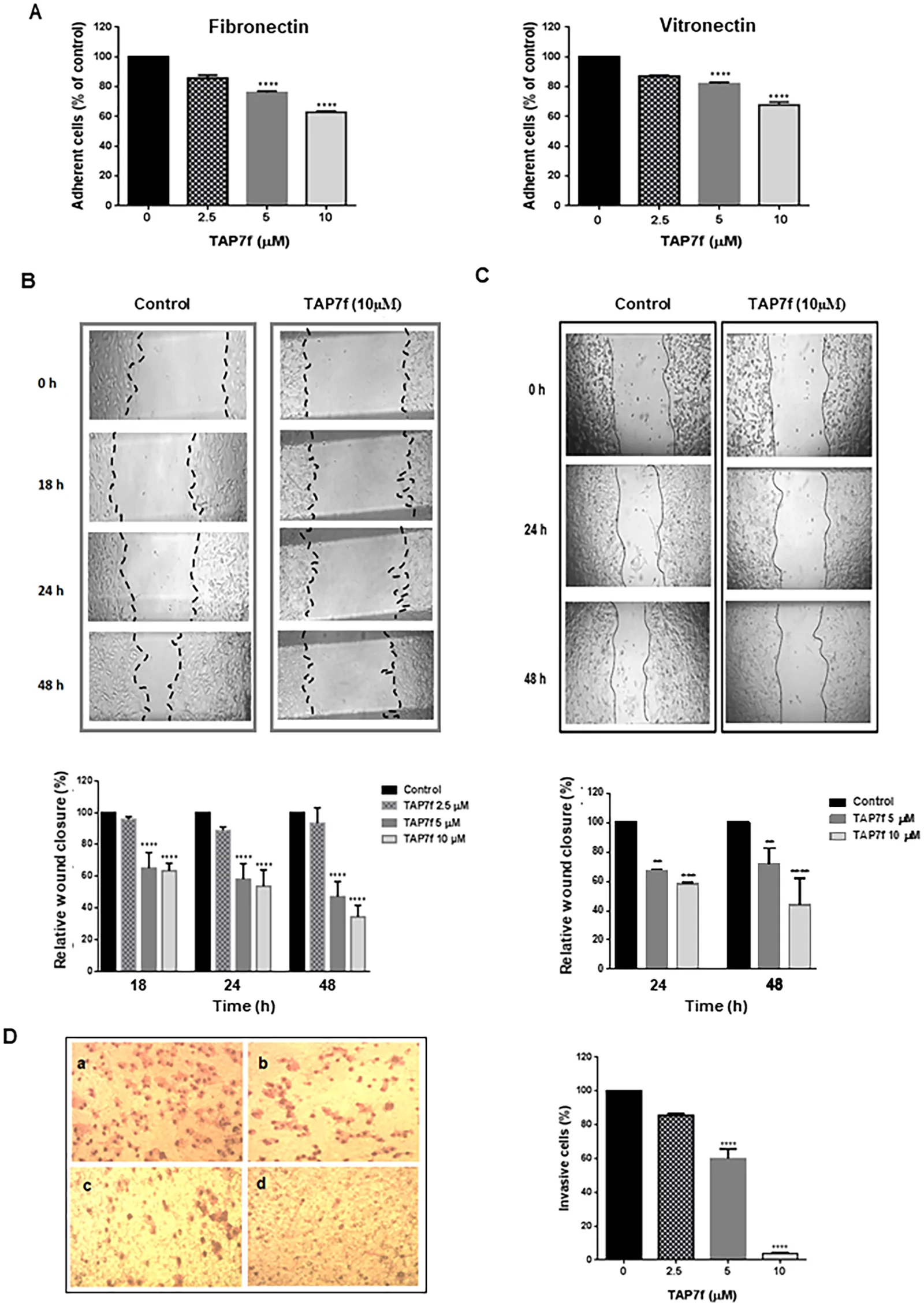
TAP7f inhibits cell adhesion, migration and invasion. **(A)** B16-F10 cells were pre-incubated with or without different concentrations of TAP7f and then added to 96-microwells previously coated with fibronectin or vitronectin. After 1 h incubation, attached cells were determined by hexosaminidase method. **(B, C)** Monolayers of B16-F10 **(B)** or A375 **(C)** cells were scratched and incubated with or without (control) different concentrations of TAP7f. Pictures were taken at different times with a camera coupled to a microscope. Photographs from one representative experiment are shown (top panels). The area of the wound was analyzed by Image J software (bottom panels). **(D)** B16-F10 cells were treated with different concentrations of TAP7f for 18 h and the invasive capacity was determined by the Transwell invasion assay. Left: representative photographs of invasive cells after incubation without (a) or with TAP7f 2.5 µM (b), 5 µM (c) and 10 µM (d). Right: quantification of invasive cells. Results are mean values ± SE of three different experiments. Statistical significance in comparison with the corresponding control values is indicated by **p < 0.01, ***p < 0.001, ****p < 0.0001.

The metastatic behavior of cancer cells is triggered by a switch in their ability to adhere to ECM or endothelial cells and to migrate and invade basement membranes and connective tissue (Lambert et al., 2017). To study TAP7f effect on melanoma cell adhesion, B16-F10 cells were pre-incubated with 2.5–10 µM of TAP7f for 18 h and allowed to adhere for 1 h to ECM proteins, such as fibronectin or vitronectin. As shown in **Figure 2A**, the pre-incubation of melanoma cells with TAP7f inhibited cell adhesion to both fibronectin and vitronectin in a concentration-dependent manner. In order to evaluate TAP7f effect on cell migration, we decided to perform wound healing assays. Results showed that a 5 µM concentration of TAP7f significantly inhibited cell migration on both, murine B16-F10 (**Figure 2B**) and human A375 cells (**Figure 2C**). The effect of TAP7f on cell invasion was determined by Transwell chamber assays. We found that the penicillin derivative inhibited B16-F10 cell invasion to the basement membrane extract Geltrex™ after 18 h in a dose-dependent manner (**Figure 2D**). It’s worth mentioning that TAP7f did not affect cell viability in the conditions employed for adhesion, migration and invasion assays (**Supplementary Figure 1**).

### TAP7f Inhibits the Activity and Expression of Metalloproteinases

**Figure 3:**
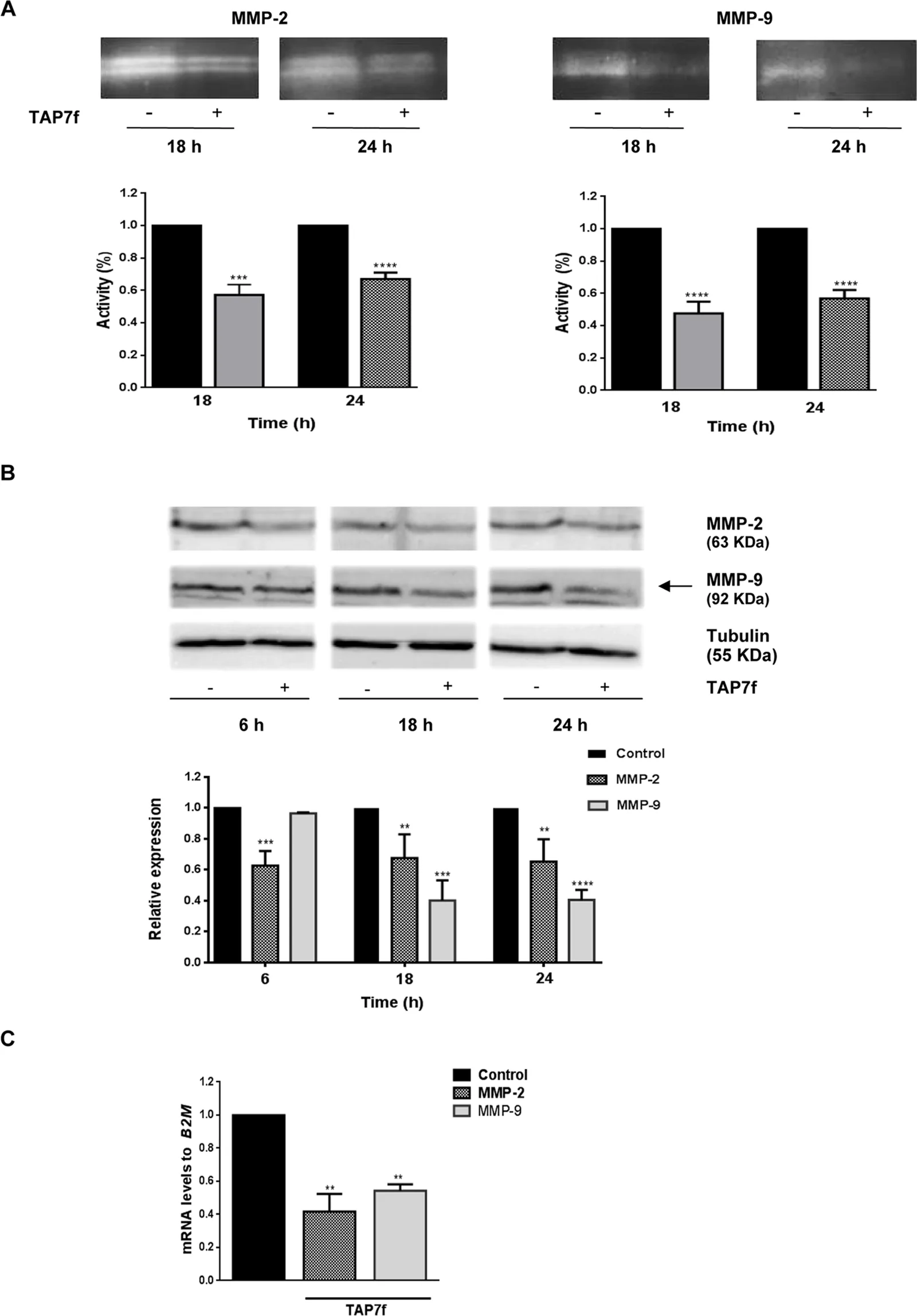
TAP7f effect on MMP-2 and -9 activity and expression levels. **(A)** B16-F10 cells were incubated for 18 or 24 h with culture medium in the presence or absence of 10 μM of TAP7f. The enzymatic activities of MMP-2 and MMP-9 were determined by gelatin zymography. One representative zymography is shown (top). Quantification was performed by densitometric analysis (bottom). **(B)** Western blot assays of cell lysates from TAP7f treated or not cells were performed with anti-MMP-2 and anti-MMP-9 antibodies. Data quantification was performed by densitometric analysis**. (C)** The mRNA expression levels of MMP-2 and MMP-9 were measured by RT-qPCR. Statistical analyses were performed by one-way ANOVA followed by Dunnett´s post-hoc tests. **p < 0.01, ***p < 0.001, ****p < 0.0001, n = 3.

Metalloproteinases, specifically MMP-2 and MMP-9, are usually involved in the extracellular matrix disruption that allows tumor cell migration and invasion. In order to study TAP7f effect on MMP-2 and -9 activity, we performed zymography assays after incubating B16-F10 cells with a 10 µM concentration of the derivative for 18 and 24 h. Results obtained showed that TAP7f significantly inhibited the activity of both metalloproteinases (**Figure 3A**). In addition, when TAP7f effect on MMPs expression was studied by western blot assays, a significant decrease in the levels of MMP-2 was observed after 6 h of incubation, remaining invariable up to 24 h (**Figure 3B**). However, reduced expression levels of MMP-9 were detected after 18 and 24 h of treatment (**Figure 3B**). We further investigated the effect of TAP7f on MMP-2 and -9 transcriptional expression by Reverse Transcription quantitative Polymerase Chain Reaction (RT-qPCR) assays. Results indicated that after 18 h of treatment with TAP7f, mRNA levels of both MMPs were significantly downregulated (**Figure 3C**).

### TAP7f Downregulates Wnt/β-Catenin Pathway in Melanoma Cells

**Figure 4:**
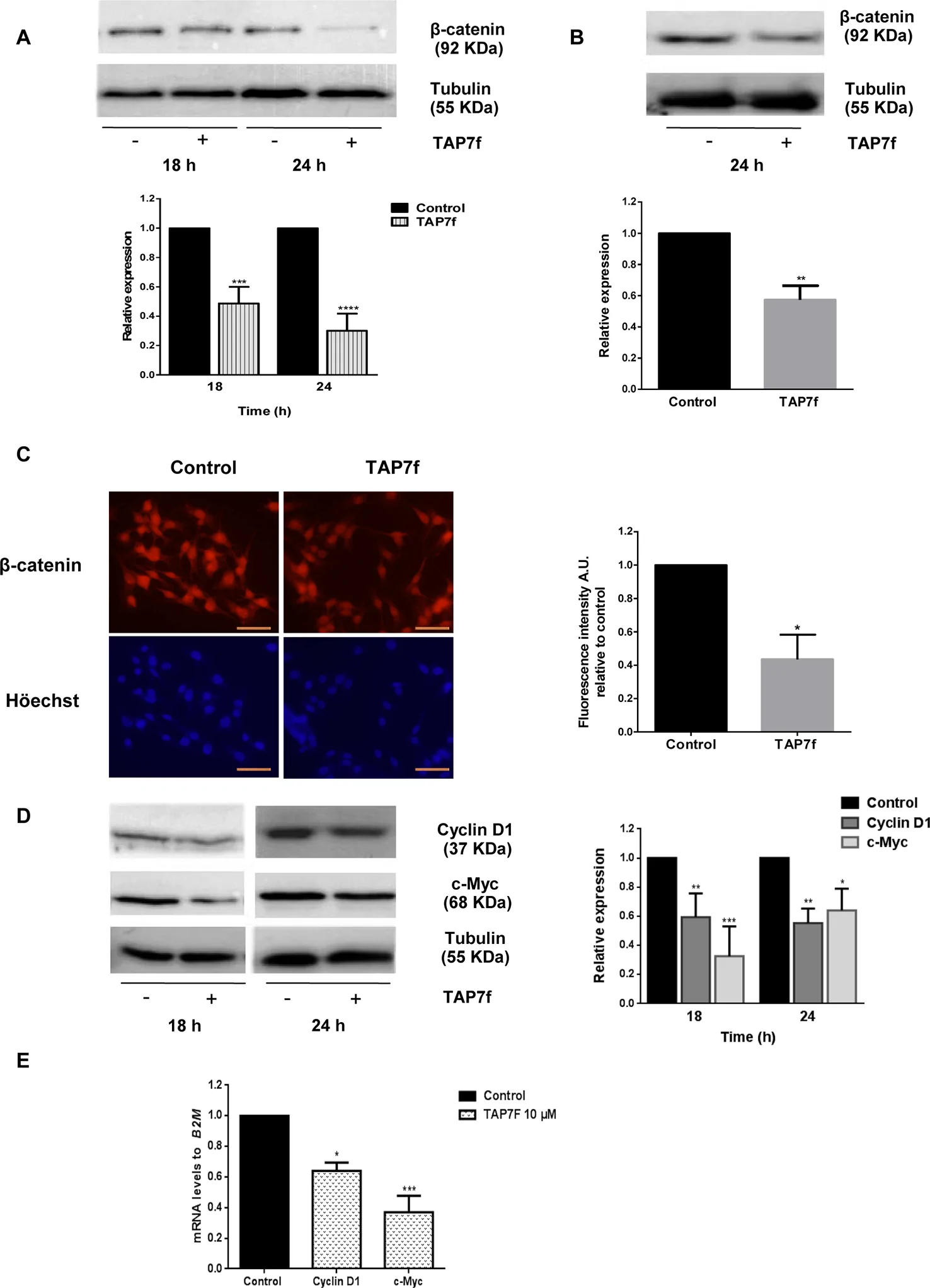
TAP7f inhibits the expression of β-catenin and downstream targets cyclin-D1 and c-Myc. B16-F10 **(A, D)** or A375 **(B)** cells were incubated in the presence or absence of 10 μM of TAP7f. Total cell lysates were processed for western blot analysis as described in *MATERIALS AND METHODS*. Results from one representative experiment are shown. Data quantification was performed by densitometric analysis. **(C)** Cells were treated for 18 h with TAP7f, followed by immunostaining for β-catenin (red) and counterstaining with Höechst 33258 (blue). Magnification 400×. Scale bar: 50 µm (left panel). Fluorescence intensity was analysed by Image J software (right panel). **(E)** The mRNA expression levels of cyclin-D1 and c-Myc were measured by RT-qPCR. Statistical analyses were performed by one-way ANOVA followed by Dunnett´s post-hoc tests **(A, D, E)** or Student’s t-test **(B, C)**. *p < 0.05, **p < 0.01, ***p < 0.001, ****p < 0.0001, n = 3.

In order to study the possible role of TAP7f in Wnt/β-catenin signaling pathway, we evaluated by western blot the expression of β-catenin in whole cell lysates obtained after incubating B16-F10 cells for 18 and 24 h and A375 cells for 24 h with a 10 µM concentration of TAP7f. Results showed a significant time-dependent decrease in the cellular expression of β-catenin both in B16-F10 (**Figure 4A**) and A375 (**Figure 4B**) cells. We also observed a reduction of β-catenin nuclear translocation by immunocytochemistry in TAP7f-treated B16-F10 (**Figure 4C**) and A375 (**Supplementary Figure 2**) cells. When we studied the protein expression levels of some β-catenin downstream targets, as cyclin-D1 and c-Myc, we found that the expression of both proteins was inhibited after 18 and 24 h of treatment with TAP7f (**Figure 4D**). Additionally, we found by RT-qPCR assays that TAP7f decreased cyclin-D1 and c-Myc transcriptional levels after 18 h of treatment (**Figure 4E**).

### TAP7f Inhibits Epithetial–Mesenchymal Transition in Melanoma Cells

**Figure 5:**
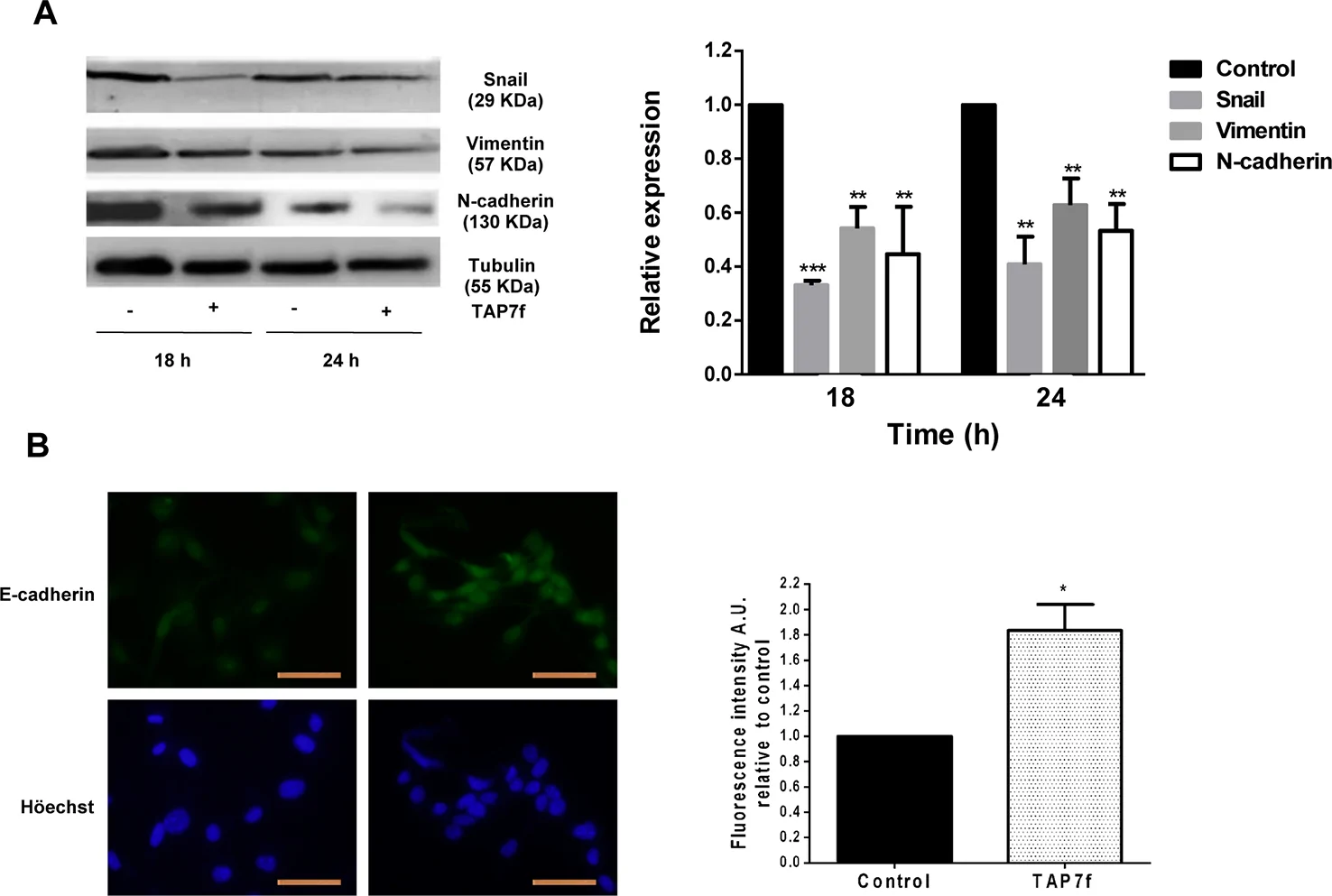
TAP7f inhibits the epithetial-mesenchymal transition. **(A)** B16-F10 cells were incubated for 18 and 24 h in the presence or absence of 10 μM of TAP7f. Total cell lysates were processed for western blot analysis as described in *MATERIALS AND METHODS*. Results from one representative experiment are shown. Data quantification was performed by densitometric analysis. **(B)** Cells were treated for 18 h with TAP7f, followed by immunostaining for E-cadherin (green) and counterstaining with Höechst 33258 (blue). Magnification 400×. Scale bar: 50 µm (left panel). Fluorescence intensity was analysed by Image J software (right panel). Statistical analyses were performed by one-way ANOVA followed by Dunnett´s post-hoc tests **(A)** or Student’s t-test **(B)**. **p < 0.01, ***p < 0.001 significantly different from non-stimulated cells, n = 3.

During EMT progression, cells acquire remarkable morphological alterations and mesenchymal properties, evidenced by an increment in the levels of mesenchymal markers such as vimentin and N-cadherin, and the reduction of epithelial markers such as E-cadherin. In addition, Snail is one of the transcription factors that play a crucial role as a molecular switch of the EMT program. Therefore, we decided to evaluate the effect of TAP7f on the expression of Snail and different EMT markers. Results obtained showed a decrease in the expression levels of Snail, vimentin and N-cadherin (**Figure 5A**) and an increment in the expression levels of E-cadherin (**Figure 5B**), after incubation of B16-F10 cells with the penicillin derivative, suggesting that TAP7f promotes the inhibition of the EMT. Furthermore, TAP7f also increased expression levels of E-cadherin in human A375 cells (**Supplementary Figure 3**).

### TAP7f Decreases Integrin αvβ3 Expression in B16-F10 Cells

**Figure 6:**
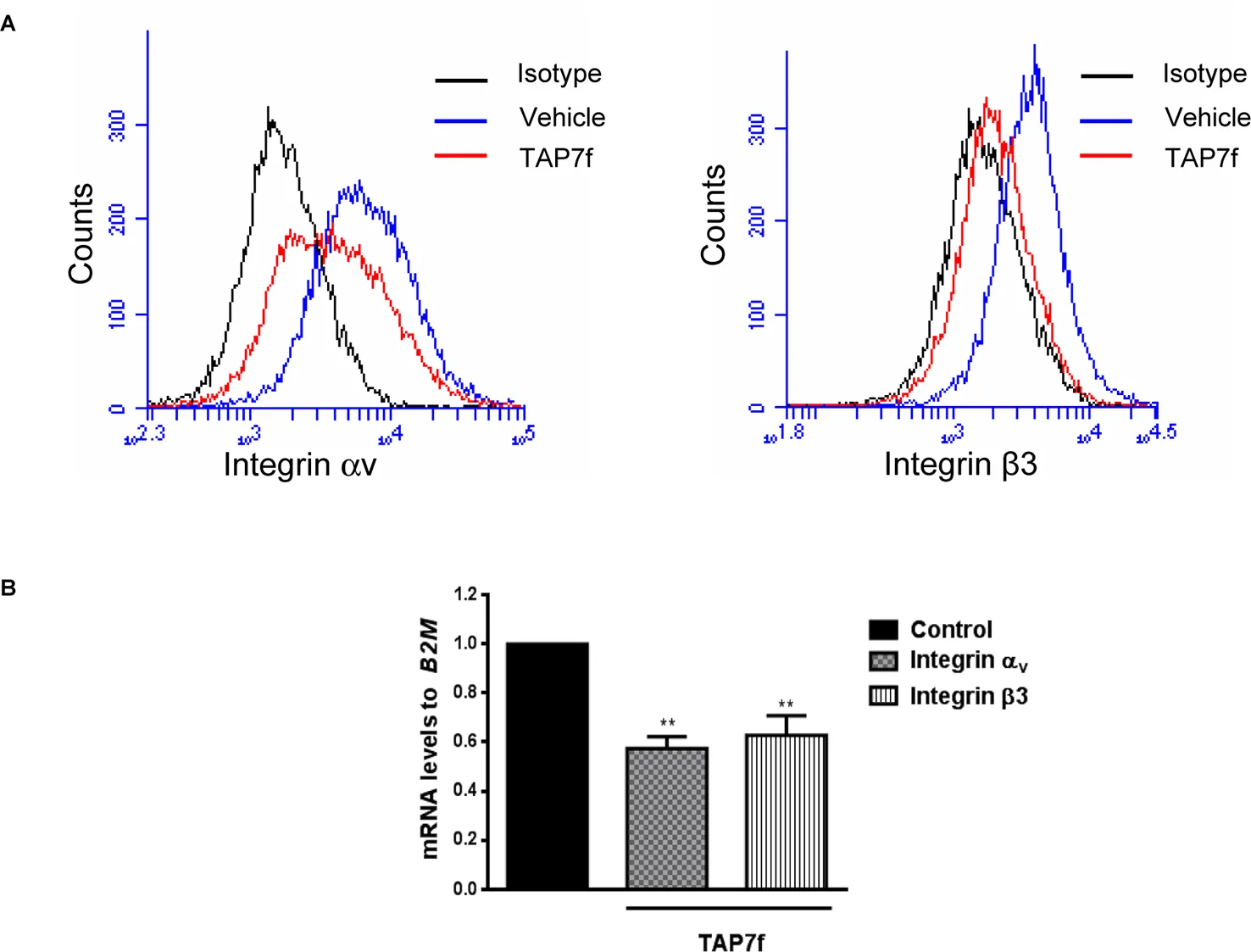
TAP7f inhibits the expression of integrin αvβ3. **(A)** B16-F10 cells pre-incubated for 24 h in the presence or absence of 10 μM of TAP7f were fixed with 1% paraformaldehyde and integrins αv and β3 expression was analyzed by flow cytometry under the conditions described in *MATERIALS AND METHODS*. **(B)** B16-F10 cells were incubated for 18 h with or without TAP7f and the mRNA expression levels of integrin αv and β3 subunits were measured by RT-qPCR. Statistical analyses were performed by one-way ANOVA followed by Dunnett´s post-hoc tests. **p < 0.01 significantly different from non-stimulated cells, n = 3.

Integrins are heterodimeric transmembrane proteins consisting of one α- and one β subunit which facilitate cell–cell and cell–extracellular matrix interactions and play major roles in a variety of cell functions, such as differentiation, adhesion and migration (Richard, 1987; Desgrosellier and Cheresh, 2010). It has been reported that an increased expression of integrin αvβ3 is associated with more invasive and metastatic potential of melanoma cells (Hsu et al., 1998; Voura et al., 2001; Huang and Rofstad, 2018). Therefore, we decided to study the effect of TAP7f on integrin αV and β3 subunits expression in B16-F10 cells. Results obtained by flow cytometry assays showed a decrease in the cell membrane expression of integrins αV and β3 after 24 h of treatment (**Figure 6A**). Accordingly, by RT-qPCR experiments, we also found a reduction of integrins αV and β3 mRNA levels induced by TAP7f (**Figure 6B**).

### *In Vivo* Anti-Metastatic Effect of TAP7f

**Figure 7:**
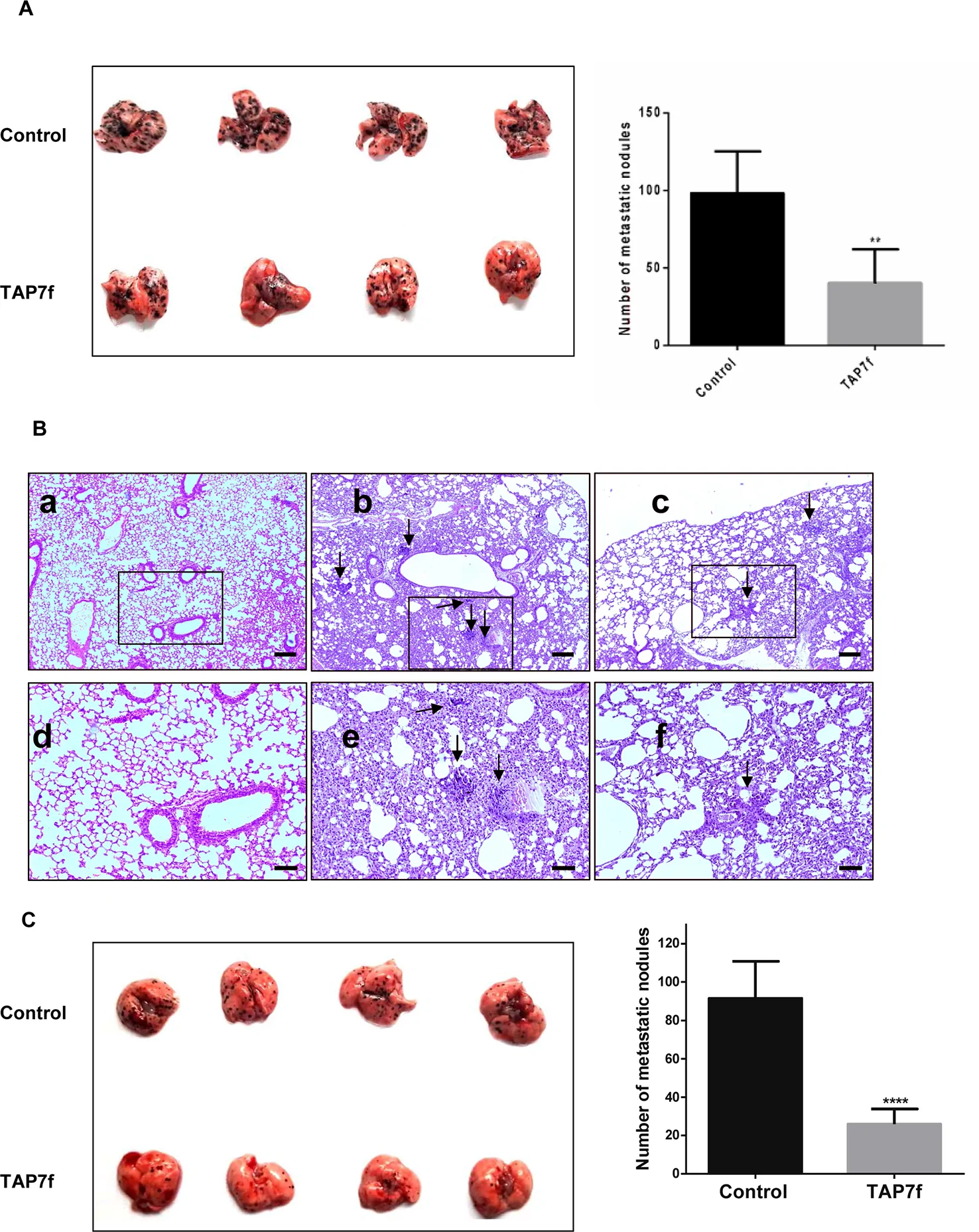
*In vivo* anti-metastatic effect of TAP7f. **(A)** B16-F10 cells pre-incubated with TAP7f or vehicle were injected (4 × 10^5^ cells) intravenously in the tail vein of each mouse. Nine days after cell inoculation mice lungs were removed and the number of nodules in each one was counted. **(B)** Lungs from mice that were not injected with cells (a,d), injected with untreated cells (b,e) or injected with TAP7f treated cells (c,f) were fixed in 10% formaldehyde in PBS and embedded in paraffin. Sections of 5 μm were then revealed by hematoxylin–eosin staining (Magnification 40×, for a,b,c, scale bar: 200 µm; 100×, for d,e,f, scale bar: 100 µm). **(C)** Mice were injected *via* tail vein with B16-F10 cells (3 × 10 ^5^ cells) and, following cell injection, were treated daily with 10 mg/kg of TAP7f or vehicle. Twelve days after tumor injection, lungs were removed and fixed. Metastastic foci at lung surfaces were photographed and counted. Results represent mean values ± SE. Statistical analysis of data was performed by using the Student’s t-test **p < 0.01, ****p < 0.0001 n = 5.

Based on the anti-metastatic potential of TAP7f described herein *in vitro*, we first explored the metastatic capacity of TAP7f pre-treated B16-F10 cells in an experimental lung metastasis model. B16-F10 cells were incubated with a 10 μM concentration of TAP7f for 18 h and injected in C57BL/6J mice through the tail vein. On day 9, mice were sacrificed for lung evaluation. As shown in **Figure 7A**, mice injected with cells that had been pre-treated with TAP7f showed a reduction of approximately 50% in the number of lung nodules. Hematoxylin and eosin stained sections of lungs from mice injected with untreated cells showed five to six tumor islands of variable sizes per lung section, whereas lungs from mice injected with TAP7f treated cells showed approximately one to two tumor islands per lung section (**Figure 7B**). In order to confirm that TAP7f inhibition of lung invasion by tumor cells was due to a prevention of the metastatic process and not to a cytotoxic effect, B16-F10 cells treated or not with TAP7f for 18h were injected subcutaneously in the right flank of C57BL/6J mice and tumor volumes were measured periodically after cell inoculation. Results obtained showed that there were no significant differences in tumor sizes between mice injected with TAP7f treated or untreated cells, suggesting that, under the experimental conditions employed for the *in vivo* metastasis model, TAP7f did not exert any antiproliferative effect (**Supplementary Figure 4**).

To further explore the anti-metastatic effect of TAP7f treatment *in vivo*, B16-F10 cells were injected into the tail vein and mice were daily treated with 10 mg/kg of TAP7f (i.p.). On day 12, mice were sacrificed for lung evaluation. Results showed that TAP7f reduced approximately 70% the number of lung nodules, compared to the vehicle (**Figure 7C**). Hematoxilin–eosin stained sections of lungs also showed a decrease in the number of tumor foci after TAP7f treatment (**Supplementary Figure 5**).

## Discussion

Important advances have improved cancer treatment over the last years, however, metastasis continues being one of the most important challenges to gain the battle against cancer. Therefore, there is an urgent need to find drugs aimed at blocking one or more steps of the metastatic cascade. In a previous work, we have examined the *in vitro* antitumor action of the non-antibiotic triazolyl penicillin derivative TAP7f in different non metastatic human and murine adenocarcinoma cell lines (Blank et al., 2018). We also demonstrated the *in vivo* efficacy of this derivative in a murine melanoma model (Blank et al., 2018). In this work, we reported for the first time the anti-metastatic properties of TAP7f against highly metastatic melanoma cells *in vitro* and *in vivo*. We evaluated TAP7f antiproliferative activity on different human and murine metastatic cell lines and we selected B16-F10 and A375 cells to further study TAP7f anti-metastatic properties. It is important to mention that TAP7f antiproliferative activity is 14 and 10 times higher against B16-F10 and A375 cells, respectively, regarding non-tumor cells, as we have previously reported an IC_50_ value of 100 µM for NMuMG mammary gland cells (Cornier et al, 2014). Murine Bl6-F10 cells were established from lung metastasis of B16-F0 cells by i.v. injection through 10 successive selections and are usually employed to investigate the anti-metastatic effects of different compounds (Fidler, 1973; Mummert et al., 2003; Chien et al., 2015; Kee et al., 2017). The interaction of melanoma cells with extracellular matrix proteins is one of the initial steps crucial for metastatic dissemination and we found that TAP7f inhibited cell adhesion to the immobilized matrix proteins vitronectin and fibronectin. This inhibition may probably be explained by TAP7f downregulation of integrin αvβ3 expression, which is known to mediate the adhesion of B16-F10 cells to vitronectin and fibronectin (Wu et al., 2001; Boettiger et al., 2001). Another important step of metastasis is the migration of cancer cells from the primary tumor into the blood or lymph system followed by their invasion into a different tissue where a secondary tumor grows (Lambert et al., 2017). We found that TAP7f inhibited migration of both B16-F10 and A375 cells in non-cytotoxic incubation conditions. Furthermore, we also demonstrated that TAP7f inhibited invasion and reduced both MMP-2 and MMP-9 expression as well as enzymatic activities in B16-F10 cells. Since both proteases have been involved in the degradation of ECM and basement membrane, our results suggest that these MMPs could play an important role in the inhibition of melanoma metastasis induced by TAP7f. Numerous research papers have reported that MMPs expression could be regulated by the Wnt/β-catenin signaling pathway (Wu et al., 2007; Ingraham et al., 2011; Qu et al., 2014; Lee et al., 2014; Chen et al., 2017). In melanoma, the exact role of Wnt/ß-catenin signaling remains controversial since some authors showed that elevated levels of β-catenin correlated with reduced melanoma cell proliferation and suppression of invasion, whereas other researchers reported that β-catenin is a key factor determining invasive capacity and tumorigenicity of primary and metastatic melanoma cell lines (Chien et al., 2009; Arozarena et al., 2011; Sinnberg et al., 2011). Our results showed that TAP7f, which inhibited melanoma cell proliferation, migration and invasion, also reduced β-catenin nuclear translocation and diminished expression levels of β-catenin as well as some of the downstream targets cyclin-D1, c-Myc and MMPs-2 and -9, supporting an invasive role for Wnt/β-catenin pathway in metastatic melanoma cells. TAP7f inhibition of this pathway could be mediated by integrin αvβ3 downregulation, since it has been reported that integrins contribute to β-catenin stabilization in melanoma cells (Piva et al, 2017). It is well known that Wnt/β-catenin, together with other signaling pathways such as Notch, Hedgehog and transforming growth factor β (TGF-β), are involved in EMT, a complex cellular process that contributes to the metastatic potential of malignant melanocytes (Caramel et al., 2013; Liu et al., 2015). Furthermore, the abnormal expression of transcription factors related to EMT is frequently observed in carcinomas (Thiery et al., 2009). In this sense, it has been reported that the zinc-finger transcription factor Snail is activated when β-catenin translocates to the nucleus and triggers the switch in gene expression from epithelial to mesenchymal phenotype (Yook et al., 2006; Wang et al., 2013). Moreover, the primary mechanism of Snail-induced EMT is the repression of the epithelial marker E-cadherin, which causes reduced cell-cell adhesion and promotes migratory capacity (Kaufhold and Bonavida, 2014). We found that TAP7f downregulated the expression of Snail and the mesenchymal markers vimentin and N-cadherin and increased expression levels of E-cadherin, suggesting that the anti-metastatic effect of this synthetic compound is also mediated by the inhibition of EMT in melanoma cells. It has been demonstrated that the integrin/FAK signaling pathway can induce EMT in melanoma cells, thus, the alteration in EMT caused by TAP7f may be triggered by the reduction of integrin αvβ3 expression levels (Hakomori, 2010; Sun et al., 2011; Ruan et al., 2012). However, we cannot discard the possibility that the observed reduction of the expression of integrins in the presence of TAP7f is a consequence of the downregulation of Snail, since it has been reported that Snail can upregulate the expression of integrin αvβ3 (Haraguchi et al., 2008). It is well documented that increased integrin αvβ3 expression plays an important role during melanoma progression, promoting cell proliferation, attachment, transendothelial migration and invasion (Felding-Habermann et al., 2002; Kuphal et al., 2005; Pisano et al., 2013; Pickarski et al., 2015). Moreover, the high expression of integrin αvβ3 in different tumor cells, including osteosarcomas, neuroblastomas, glioblastomas, melanomas, lung carcinomas, and breast cancer, has made it a molecular target for the development of many anticancer drugs which are currently being tested in clinical trials (Zitzmann et al., 2002; Raab-Westphal et al., 2017). The specific mechanisms that regulate TAP7f inhibition of integrin αvβ3 expression levels are now under study.

**Figure 8:**
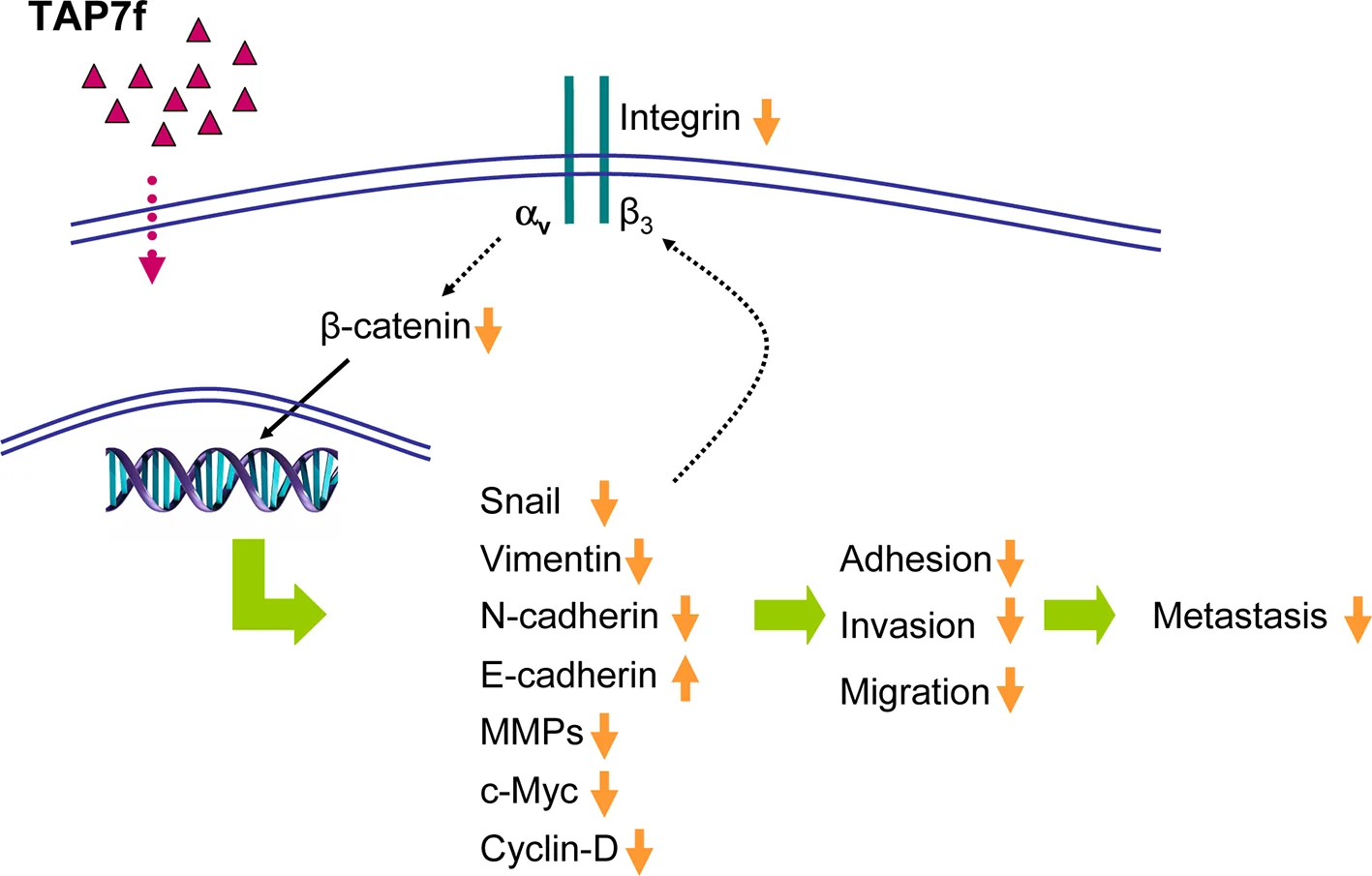
Proposed mechanisms for TAP7f anti-metastatic effect on murine melanoma cells. TAP7f-induced downregulation of integrin αvβ3 expression may inhibit Wnt/β-catenin pathway and epithelial mesenchymal transition, leading to the inhibition of adhesion, invasion and migration. The reduced expression of Snail may also contribute to the decrease of integrin αvβ3 expression.

In conclusion, we have demonstrated that TAP7f, previously characterized as a novel penicillin derivative with antitumor properties *in vitro* and *in vivo*, can also inhibit melanoma cell adhesion, migration and invasion, and the molecular mechanisms involved in the metastatic process (**Figure 8**). Moreover, we found that TAP7f diminished melanoma lung metastasis *in vivo*, suggesting that this penicillin derivative may be considered as a potential anti-metastatic agent for the treatment of melanoma.

## Data Availability Statement

The datasets generated for this study are available on request to the corresponding author.

## Ethics Statement

The animal study was reviewed and approved by the Institutional Animal Care and Use Committee (CICUAL) of the School of Pharmacy and Biochemistry, University of Buenos Aires.

## Author Contributions

Conceived and designed the experiments: EB, LR, and VB. Performed the experiments: mainly EB, FC (PCR and flow cytometry experiments), LR, and VB (*in vivo* experiments). Analyzed the data: EB, LR, and VB. Contributed reagents/material/analysis tools: LR, VB, FC, GC, PC, DB, CD, and EM. Wrote the paper: VB and LR. All authors reviewed and approved the manuscript before submission.

## Funding

This work was supported by funds from Consejo Nacional de Investigaciones Científicas y Técnicas (CONICET, PIP 0154: “Propiedades y mecanismo de acción de nuevos agentes antitumorales: péptidos quiméricos del IFN alfa, ftalocianinas de Zn(II) y derivados sintéticos de penicilinas”), Universidad de Buenos Aires (Programación Científica 2014-2017, UBACYT 20020130100024: “Mecanismos de acción de moléculas que intervienen en procesos que regulan la proliferación celular: rol de citoquinas y nuevos agentes antitumorales”), INC nº 21439671 and Agencia Nacional de Promoción Científica y Tecnológica (PICT 2017/1278 and PICT 2015/0874). PC, DB, CD, and EM also thank CONICET (PUE-IQUIR 2016), Agencia Nacional de Promoción Científica y Tecnológica (PICT 2694/2654) and Universidad Nacional de Rosario (BIO 514) for financial support.

## Conflict of Interest

The authors declare that the research was conducted in the absence of any commercial or financial relationships that could be construed as a potential conflict of interest.
