# Correction: A penicillin derivative exerts an anti-metastatic activity in melanoma cells through the downregulation of integrin αvβ3 and Wnt/β-catenin pathway

**DOI:** 10.3389/fphar.2026.1943341

**Published:** 2026-09-29

**Authors:** Elizabeth Barrionuevo, Florencia Cayrol, Graciela A. Cremaschi, Patricia G. Cornier, Dora B. Boggián, Carina M. L. Delpiccolo, Ernesto G. Mata, Leonor Roguin, Viviana C. Blank

**Affiliations:** 1 Laboratorio de Oncología y Transducción de Señales, Instituto de Química y Fisicoquímica Biológicas (IQUIFIB), Departamento de Química Biológica, Facultad de Farmacia y Bioquímica, Universidad de Buenos Aires, CONICET, Buenos Aires, Argentina; 2 Laboratorio de Neuroinmunomodulación y Oncología Molecular, Instituto de Investigaciones Biomédicas, Facultad de Ciencias Médicas, Pontificia Universidad Católica Argentina (UCA), CONICET, Buenos Aires, Argentina; 3 Laboratorio de Química Orgánica, Instituto de Química Rosario (CONICET-UNR), Facultad de Ciencias Bioquímicas y Farmacéuticas, Universidad Nacional de Rosario, Rosario, Argentina

**Keywords:** triazolylpeptidyl penicillin, anti-metastatic effect, murine melanoma, β-catenin, metalloproteinases-2 and -9, integrin αvβ3

There was a mistake in Figure 2B as published. The mistake was made when inserting the images for the migration experiments. This is likely due to the large number of similar images we handled. Additionally, the boundary line marking cell front in the top-left panel was inadvertently shifted, possibly during an ungrouping process. The corrected Figure 2 appears below.

**FIGURE 2:**
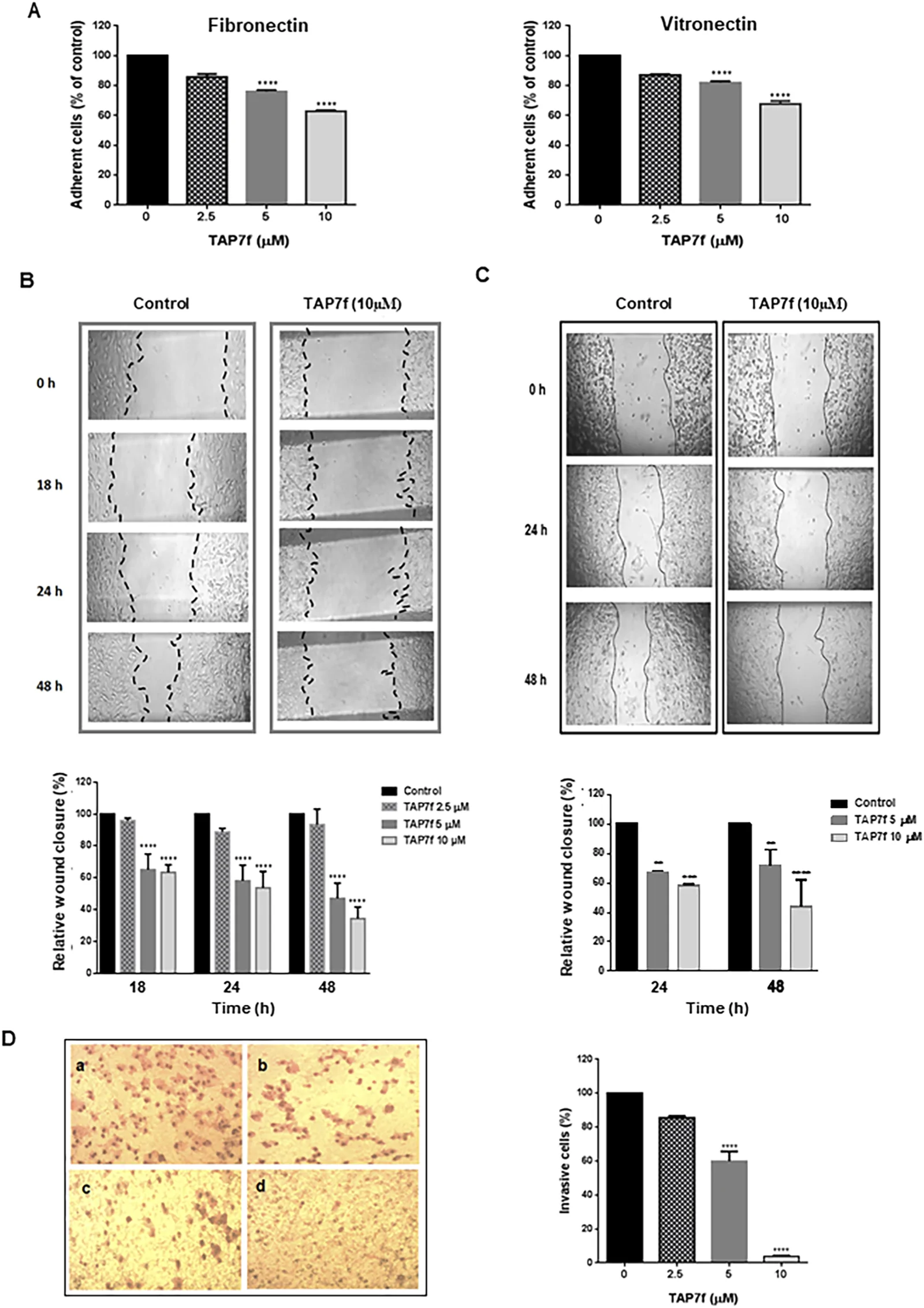
TAP7f inhibits cell adhesion, migration and invasion. **(A)** B16-F10 cells were pre-incubated with or without different concentrations of TAP7f and then added to 96-microwells previously coated with fibronectin or vitronectin. After 1 h incubation, attached cells were determined by hexosaminidase method. **(B,C)** Monolayers of B16-F10 **(B)** or A375 **(C)** cells were scratched and incubated with or without (control) different concentrations of TAP7f. Pictures were taken at different times with a camera coupled to a microscope. Photographs from one representative experiment are shown (top panels). The area of the wound was analyzed by ImageJ software (bottom panels). **(D)** B16-F10 cells were treated with different concentrations of TAP7f for 18 h and the invasive capacity was determined by the Transwell invasion assay. Left: representative photographs of invasive cells after incubation without (a) or with TAP7f 2.5 μM (b), 5 μM (c) and 10 HM (d). Right: quantification of invasive cells. Results are mean values ± SE of three different experiments. Statistical significance in comparison with the corresponding control values is indicated by **p < 0.01, ***p < 0.001, ****p < 0.0001.

The original version of this article has been updated.

