# Supplementary figures and images for: A Penicillin Derivative Exerts an Anti-Metastatic Activity in Melanoma Cells Through the Downregulation of Integrin αvβ3 and Wnt/β-Catenin Pathway
Source: Front Pharmacol. 2020 Feb 25;11:127. doi: 10.3389/fphar.2020.00127 (PMC7052307; doi:10.3389/fphar.2020.00127)

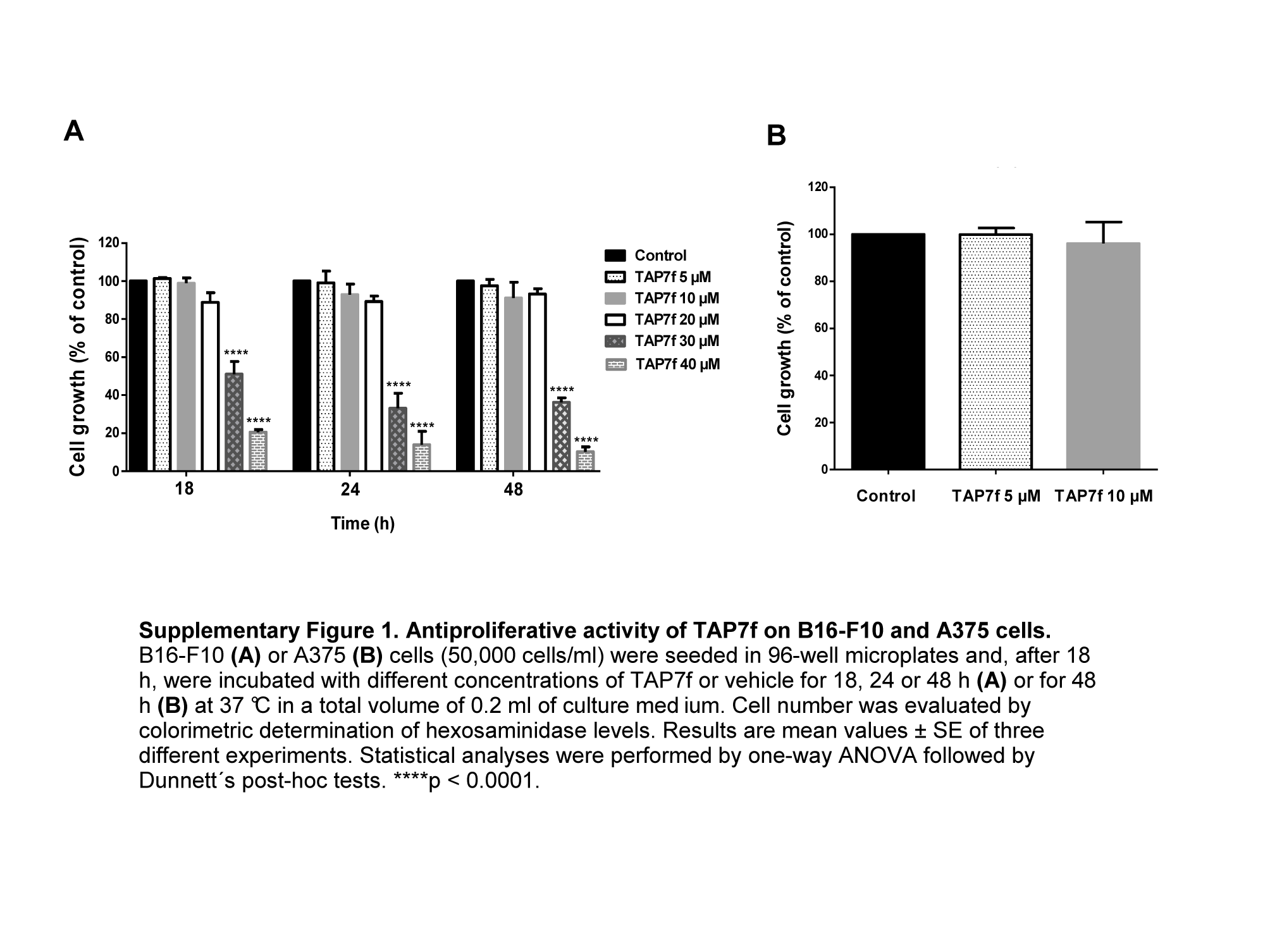

Supplement: Supplementary file 1 [file Image_1.tif]

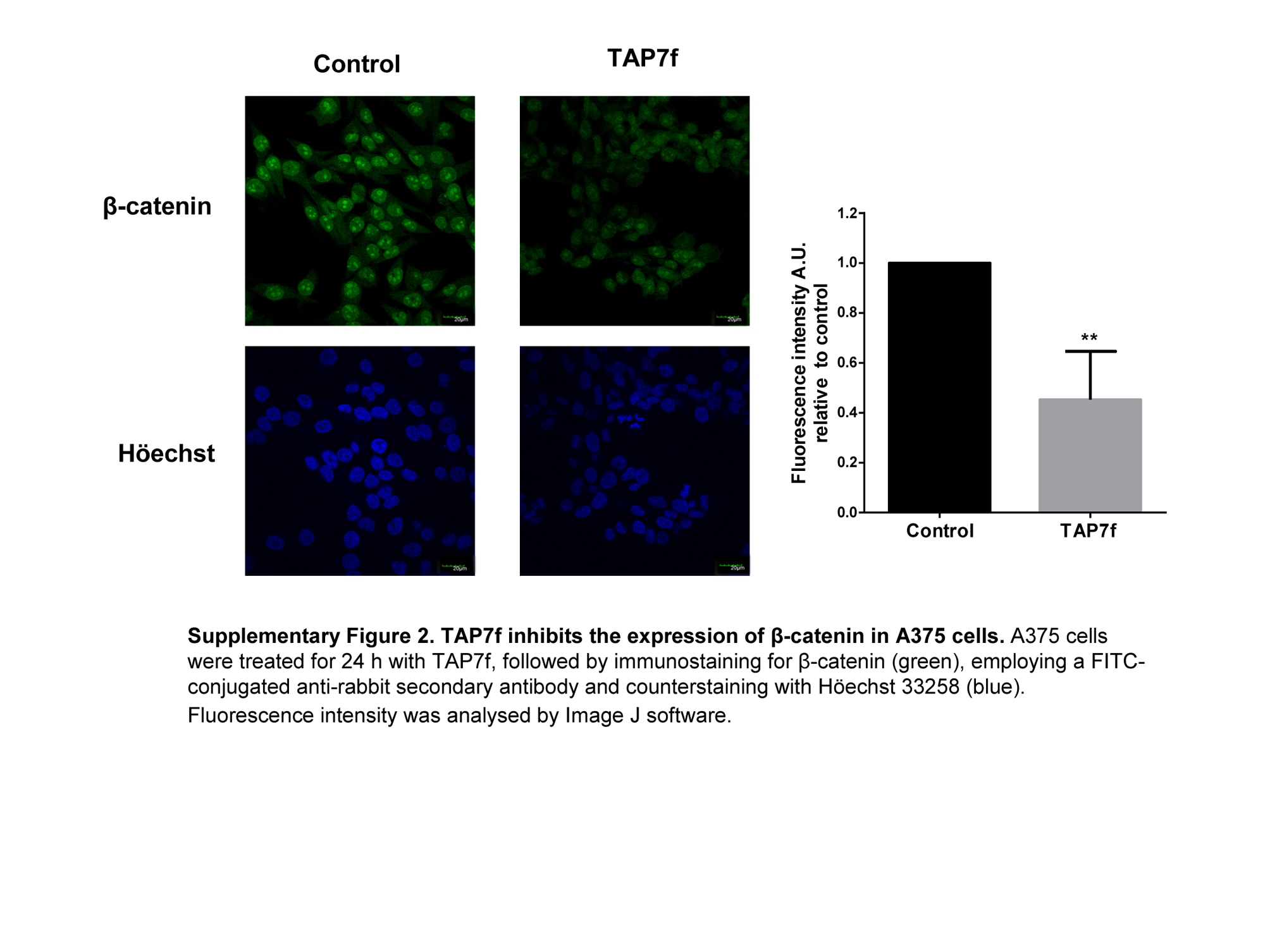

Supplement: Supplementary file 2 [file Image_2.tif]

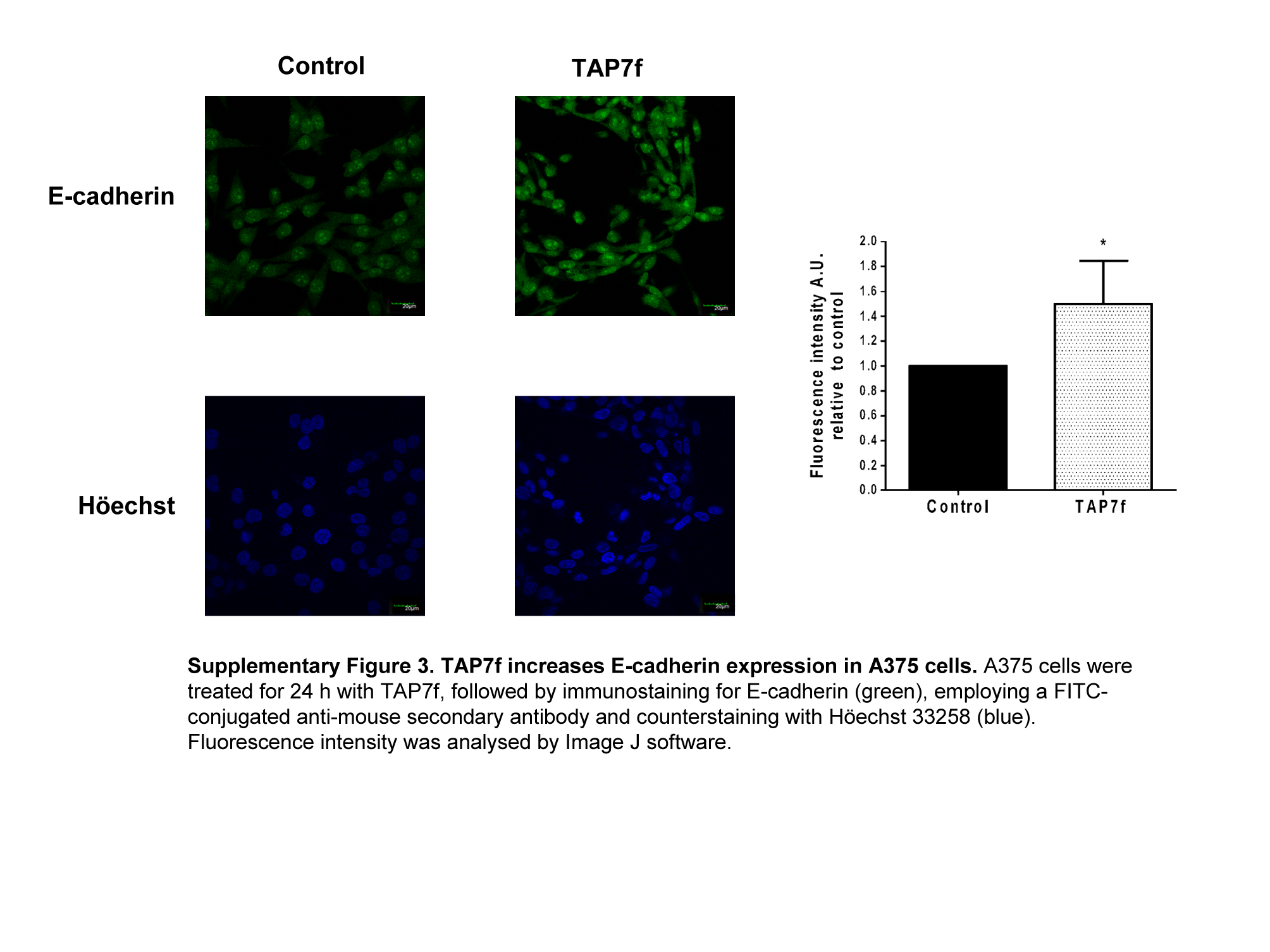

Supplement: Supplementary file 3 [file Image_3.tif]

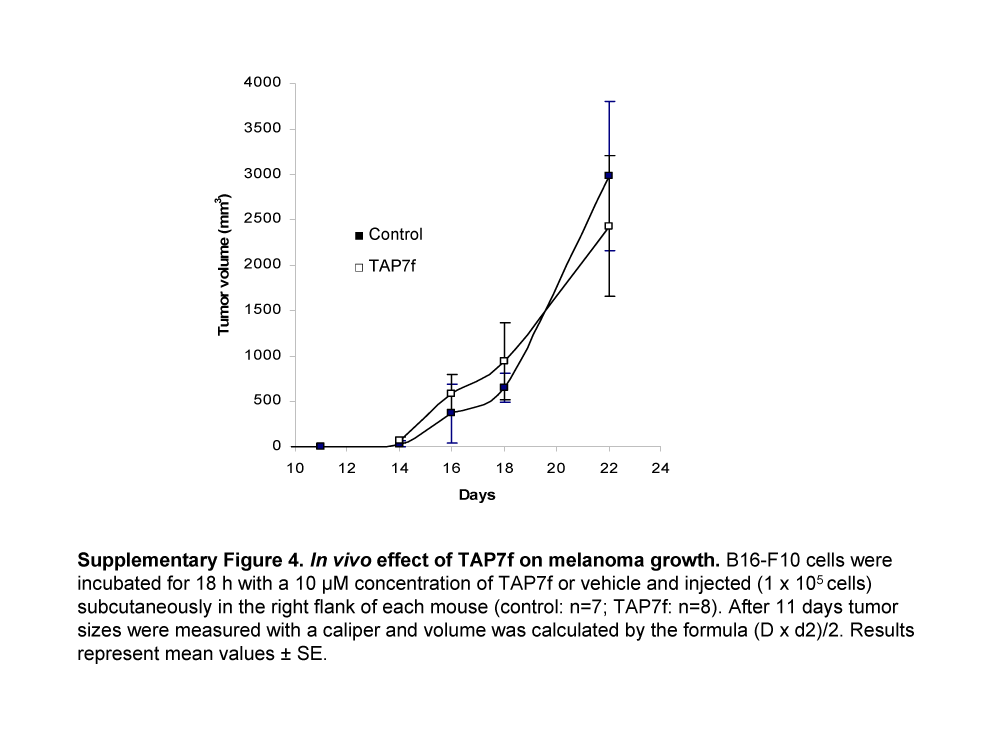

Supplement: Supplementary file 4 [file Image_4.tif]

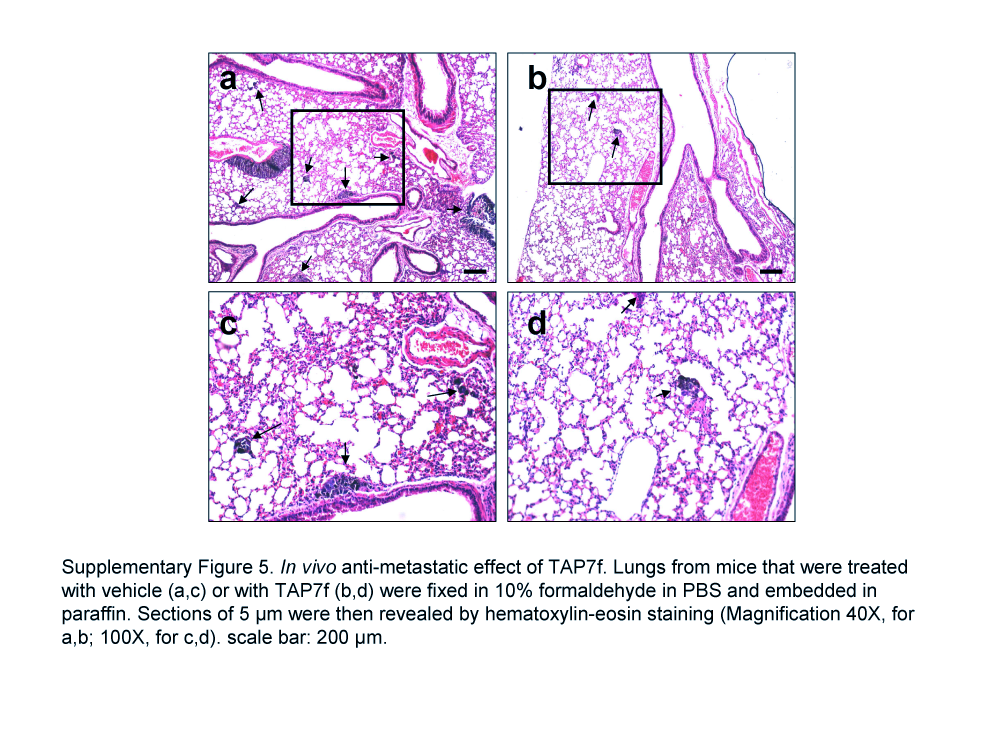

Supplement: Supplementary file 5 [file Image_5.tif]
